# The Role of Angiogenetic Factors in Preeclampsia

**DOI:** 10.3390/ijms262110431

**Published:** 2025-10-27

**Authors:** Angeliki Papapanagiotou, Maria Anastasia Daskalaki, Antonios N. Gargalionis, Angeliki Margoni, Aikaterini Domali, George Daskalakis, Athanasios G. Papavassiliou

**Affiliations:** 1 Department of Biological Chemistry, Medical School, National and Kapodistrian University of Athens, 11527 Athens, Greece; angeliki.margoni@gmail.com (A.M.); papavas@med.uoa.gr (A.G.P.); 2 Department of Obstetrics and Gynecology, “Alexandra” General Hospital, Medical School, National and Kapodistrian University of Athens, 11528 Athens, Greece; anastasia.daskalaki00@gmail.com (M.A.D.); kdomali@yahoo.gr (A.D.); gdaskalakis@yahoo.com (G.D.); 3 Laboratory of Clinical Biochemistry, “Attikon” University Hospital, Medical School, National and Kapodistrian University of Athens, 12462 Athens, Greece; agargal@med.uoa.gr

**Keywords:** preeclampsia, angiogenetic factor, VEGF, PlGF, Sflt-1, sFLT-1/PlGF

## Abstract

Preeclampsia (PE) occurs in approximately 2–8% of all pregnancies worldwide and represents one of the primary causes of maternal and fetal morbidity and mortality. Angiogenic growth factors such as placental growth factor (PlGF) and vascular endothelial growth factor (VEGF), along with their tyrosine kinase receptor (Flt-1), play a central role in placental and fetal development. Impaired placentation results in the excessive release of the antiangiogenic soluble fms-like tyrosine kinase-1 (sFlt-1) which is pivotal in the pathogenesis of PE. By binding to and neutralizing angiogenic factors, sFlt-1 disrupts normal angiogenic signaling, creating an imbalance that is often detectable before clinical symptoms of PE appear. Recent studies have highlighted the prognostic potential of the sFlt-1/PlGf ratio as an early indicator of PE risk, since this ratio has demonstrated value in both confirming and excluding PE in the high-risk population. Its incorporation into routine medical care has the potential to reduce unnecessary hospital admissions, intensive management, and premature deliveries, ultimately lowering healthcare costs. The objective of this review is to highlight the clinical utility of the sFlt-1/PlGf ratio in the prediction, diagnosis, and management of preeclampsia and to emphasize the cost-effectiveness of implementing sFlt-1/PlGF ratio measurement in the care of women at risk of developing PE.

## 1. Introduction

The placenta is a temporary organ that supports fetal growth by enabling nutrient exchange, immune protection, hormone production, and defense against harmful exposures [1].

Placental dysfunction may lead to various pathological disorders such as preeclampsia (PE), fetal growth restriction (FGR) and placental abruption, all of which are related to increased fetal/neonatal morbidity and mortality [2]. Therefore, a very significant part of obstetric care is the maintenance of placental health during pregnancy.

PE is a hypertensive disorder of pregnancy that typically appears after the 20th week and very often leads to multi-organ dysfunction [3]. It is the most dangerous form of placental dysfunction affecting 2–8% of all pregnant women worldwide. It represents one of the principal causes of maternal and fetal mortality and morbidity, being responsible for more than 70,000 maternal deaths and 500,000 fetal deaths worldwide every year [4,5]. In low-income countries, the rate is higher due to suboptimal perinatal care. In Africa and Asia, the rate of maternal deaths is about 9%, while in Latin America and the Caribbean, it represents approximately 26% of maternal mortality [6]. In the United States, it is the main cause of maternal death, severe maternal morbidity, maternal intensive care admissions, cesarean section and prematurity. Τhe increased incidence in the U.S.A is due to a rise in predisposing factors such as chronic hypertension, diabetes and obesity [7]. Moreover, women with a previous occurrence of preeclampsia face nearly twice the lifetime risk of developing cardiovascular diseases, such as heart failure, coronary artery disease, cardiovascular death and stroke, compared with those who experienced uncomplicated pregnancies, even after adjusting for potential confounders. This increased risk has been documented as early as within the first three years postpartum and remains significant for more than ten years of follow-up [8]. The aim of this review is to emphasize the clinical value of the sFlt-1/lGF ratio in predicting, diagnosis and managing PE, and to underline the cost-effectiveness of incorporating the sFlt-1/PlGF ratio into the care of women at risk for developing PE.

### 1.1. Definition of Preeclampsia

The definition of PE includes new-onset hypertension in a pregnant woman after the 20th gestational week, in conjunction with proteinuria [albumin to creatinine ratio (ACR) at least 30 mg/mmol or albuminuria at least 300 mg/24 h], or end organ malfunction. It may also present in the absence of proteinuria if there is evidence of hematological disturbances, or other organs like liver, kidneys, the brain, eyes, and placenta failure. A high blood pressure (BP) is considered a BP ≥ 140/90 mmHg on two different measurements four hours apart, while proteinuria involves protein levels above 300 mg in a 24 h urine specimen collection [9].

### 1.2. Risk Factors of Preeclampsia

The main risk factors for developing PE are PE in a previous pregnancy, chronic hypertension, diabetes, autoimmune disease (e.g., lupus antiphospholipid syndrome) and obesity (BMI > 30). Other risk factors include maternal age above 35 years, first pregnancy and multiple gestation (Table 1) [10,11,12,13,14,15,16].

## 2. Pathogenesis of Preeclampsia

The investigation of the pathophysiology of preeclampsia is an active area of international research, as the mechanisms that contribute to its development are poorly understood. The condition is attributed to a complex interaction between fetal and maternal factors, involving multiple organ systems [17]. Abnormal trophoblast migration marks the early onset of the syndrome, occurring before the woman’s awareness of pregnancy and the development of clinical symptoms [18].

PE develops in two stages: (1) abnormal placentation early in the first trimester of pregnancy, followed by (2) maternal syndrome in the late second and third trimesters, characterized by excessive antiangiogenic factor production [19,20].

The primary underlying cause of preeclampsia has been proven to be the abnormal placentation. During normal pregnancy, villous cytotrophoblast invades the inner third of the myometrium, causing the restructuring of the spiral arteries and elimination of the muscular (media) layer of the spiral arteries, thus facilitating enhanced blood flow to the placenta. This trophoblastic invasion extends deep into the arteries, transforming maternal spiral arterioles into high-capacity, high-flow vessels [21,22]. The main members of interest in placentation are VEGF-A and sFlt-1 [23].

VEGF-A is a pivotal regulator of angiogenesis, promoting endothelial cell proliferation, migration, and vascular permeability, and is essential for vascular development and homeostasis. Its expression is tightly regulated under physiological conditions such as wound healing and the menstrual cycle, ensuring a balance between pro- and anti-angiogenic factors [24]. VEGF exerts its effects through binding to endothelial receptors VEGFR1 and VEGFR2, triggering intracellular signaling cascades that drive neovascularization [25].

At the transcriptional level, VEGF-A undergoes alternative splicing, giving rise to several isoforms with distinct and sometimes opposing functions. The major pro-angiogenic variants—*VEGF121*, *VEGF165*, and *VEGF189*—bind to VEGFR1 and VEGFR2, leading to the activation of downstream kinases and gene expression programs that promote angiogenesis [26]. In contrast, the anti-angiogenic isoforms *VEGF165b* and *VEGF121b* are generated through alternative splicing events that modify their molecular structure and biological activity. The inclusion of exon 9b in *VEGF165b* results in an altered C-terminal sequence that prevents VEGFR2 activation, whereas *VEGF121b* lacks the heparin-binding domain due to its shorter sequence, allowing greater diffusibility within the extracellular matrix. Both isoforms are unable to initiate angiogenic signaling cascades and instead function as natural inhibitors of the pro-angiogenic VEGF-A family [27,28].

In PE, however, the cytotrophoblastic tissue of the placenta is unable to penetrate the uterine spiral arteries satisfactorily and displace the myoelastic structures. Thus, the remodeling of the spiral arteries fails, arteries do not dilate optimally, placental blood supply is compromised, and the abnormal development of the placenta results in poor blood supply [29]. Insufficient spiral arterial remodeling results in the narrowing of maternal vessels and associated placental ischemia [30]. These constricted spiral arteries are also susceptible to atherosclerotic changes, including lipid-laden macrophages in the lumen, fibrotic arterial wall necrosis, and mononuclear perivascular infiltrates, which exacerbate the reduction in placental perfusion [31].

The insufficient invasion of spiral arteries during PE leads to intermittent hypoxia/reoxygenation which induces oxidative stress, marked by the increased expression and activity of reactive oxygen species (ROS)-generating enzyme. Moreover, oxidative stress promotes the transcription of soluble fms-like tyrosine kinase-1 (sFlt-1) [32,33]. In addition, under hypoxic conditions, dysregulation of hypoxia inducible factors (HIF-1a and HIF-2a) prevents the proper differentiation and invasion of trophoblasts, leading to the inadequate remodeling of spiral arteries, increased sFlt-1 production, and endothelial dysfunction [19,34,35]. Both HIF-1a and HIF-2a are highly expressed in the placenta of women with preeclampsia and their overexpression [35] in experimental models has been shown to result in impaired trophoblast differentiation, proteinuria, hypertension and intrauterine growth restriction (IUGR) [36].

**Figure 1:**
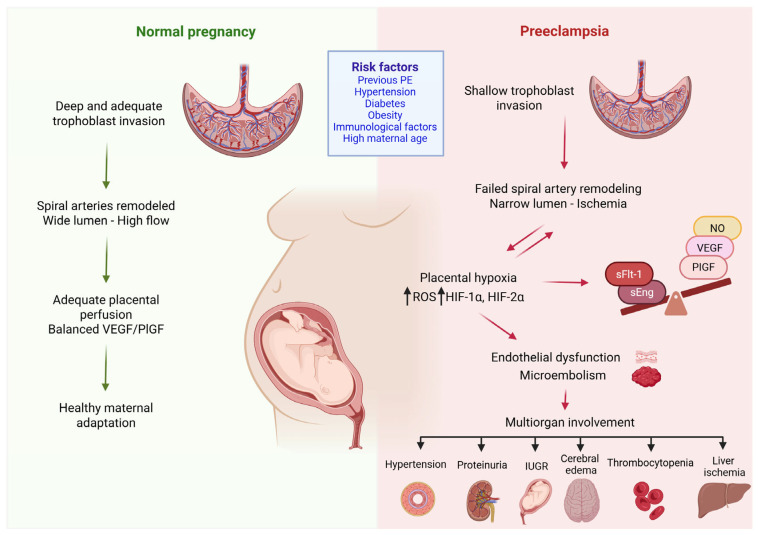
Pathogenesis of preeclampsia. Preeclampsia arises from abnormal placental development due to insufficient trophoblast invasion of the spiral arteries, resulting in a narrowed lumen and placental ischemia. The ensuing hypoxia triggers oxidative stress, characterized by increased expression of ROS-generating enzymes and the dysregulation of hypoxia-inducible factors (HIF-1a and HIF-2a). These alterations promote the excessive production of sFlt-1, leading to endothelial dysfunction and microthrombi formation. The consequence is maternal disease, driven by inadequate vascular adaptation across multiple organ systems. ROS: reactive oxygen species; sFlt-1: soluble fms-like tyrosine kinase-1, NO: nitric oxide, VEGF: vascular endothelial growth factor, PlGF: platelet growth factor, sEng: soluble endoglin, IUGR: intrauterine growth restriction, HIF-1α: hypoxia inducible factor-1α, HIF-2α: hypoxia inducible factor-2α, ↓: has as consequence. (Created with Biorender.com).

The resulting placental ischemia leads to the dysregulated production of antiangiogenic proteins, such as sFlt-1 and soluble endoglin (sEng), into the maternal circulation, causing inflammation, endothelial dysfunction, and systemic maternal disease [37,38,39]. These factors mediate subsequent reactions, resulting in endothelial dysfunction, vasoconstriction, oxidative stress and microembolism. In turn, these processes contribute to the involvement of multiple organs, causing inadequate vascular adaptation in multiple organ systems, mainly the liver, kidneys and cardiovascular system and thus leading to the clinical characteristics of PE [40,41]. Endothelial damage further leads to a reduced production of vasodilator substances, such as prostacyclin and nitric oxide, disrupting the balance between prostacyclin and thromboxane [42]. Increased thromboxane production together with decreased nitric oxide (NO) production promote platelet adhesion to the surface of the trophoblast, resulting in thrombus formation within the villi and an even greater impairment of fetal hemostasis [43]. Dysfunctional endothelial cells also produce large amounts of endothelin-1 which inactivates NO (Figure 1) [44].

Immunological abnormalities contribute to the phenotype of PE. Normally, helper T cells shift towards an anti-inflammatory Th2 phenotype; in PE, however, they shift towards a Th1 phenotype, increasing the release of proinflammatory cytokines such as interleukin 12 (IL-12) and decreasing interleukin 10 (IL-10), which leads to apoptosis and reduced trophoblast invasion [45].

The involvement of the complement system and its overactivation in preeclampsia (PE) has attracted considerable attention, as under normal conditions complement activity is suppressed during pregnancy to maintain maternal immune tolerance and support fetal development [46]. In PE, regulatory mechanisms appear insufficient, leading to excessive terminal complement activation, inflammation, and damage to the utero–placental unit, ultimately impairing placental perfusion [46,47]. The terminal complement complex C5b-9 is frequently detected in the urine and placental tissues of preeclamptic women, while earlier complement components are also observed, indicating the complement cascade’s role in disease pathogenesis [47,48,49]. Concurrently, PE and HELLP syndrome are associated with hemostatic disturbances and thrombotic microangiopathies (TMAs), characterized by elevated von Willebrand factor antigen (vWFAg) levels and reduced ADAMTS13 activity [48,50]. ADAMTS13 genetic polymorphisms may cause partial deficiency, particularly when combined with complement gene mutations or additional triggers, suggesting overlapping pathophysiological mechanisms with TMAs. Measuring ADAMTS13 activity aids in differentiating PE from other TMAs, such as thrombotic thrombocytopenic purpura (TTP) and hemolytic–uremic syndrome (HUS), and provides insight into endothelial dysfunction, prognosis, and therapeutic decision-making [51].

Moreover, several studies have demonstrated that impaired decidualization—namely, the inadequate stromal transformation of the endometrium—may contribute to the development of PE. A defective placenta may result from a combination of factors affecting both trophoblast function and decidual integrity [37], (Table 2).

## 3. Angiogenic Factors and Their Receptors

The proper formation and maintenance of the placental vascular endothelium and the placental angiogenic balance depend on the interactions involving angiogenic factors and their corresponding receptors [38]. VEGF and PlGF, members of the VEGF family, possess angiogenic functions, while sFlt-1 and soluble endoglin (sEng) exhibit antiangiogenic activities [52]. The interactions of angiogenic factors with their receptors are essential for the development and maintenance of placental vasculature as well as for preserving placental angiogenic homeostasis [23,52,53].

The vascular endothelial growth factor (VEGF) family is a large group of signaling proteins that includes VEGF (or VEGF-A), VEGF-B, VEGF-C, VEGF-D and placental growth factor (PlGF) [37,38]. Members of the VEGF family interact with one or more receptor tyrosine kinases as well as with specific co-receptors. Almost all known cellular effects of VEGF-A are mediated by vascular endothelial growth factor receptor 2 (VEGF-R2) [36,39]. The function of vascular endothelial growth factor receptor 1 (VEGFR1) is less well understood, but it likely acts by modifying VEGFR2 function, while also serving as a “pseudoreceptor”, preventing the binding of VEGF to VEGFR2 [53,54,55,56].

Angiogenic signaling plays a pivotal role in orchestrating placental morphogenesis and trophoblast differentiation, ensuring the establishment of an efficient maternal–fetal interface [57]. During early gestation, low oxygen tension induces hypoxia-inducible factor-1α (HIF-1α), which upregulates the expression of key angiogenic mediators such as vascular endothelial growth factor (VEGF) and placental growth factor (PlGF) [58,59,60]. These molecules act through their receptors, VEGFR-1 and VEGFR-2, to stimulate endothelial proliferation, migration, and vascular remodeling within the developing villous tree [38]. VEGF-driven signaling influences trophoblast lineage specification, promoting cytotrophoblast proliferation and differentiation into invasive extravillous trophoblasts that remodel the maternal spiral arteries. Trophoblast differentiation begins at the time of implantation, when cytotrophoblasts give rise to two main subpopulations: the multinucleated syncytiotrophoblasts and the invasive extravillous trophoblasts (EVTs). Early lineage specification is tightly regulated by signaling cascades such as the Wnt/β-catenin and bone morphogenetic protein (BMP) pathways, which drive cellular proliferation, polarization, and differentiation. In parallel, the Notch signaling pathway—particularly the activity of Notch1 and its ligand Delta-like 4 (DLL4)—plays a crucial role in fine-tuning trophoblast invasion and the formation of the syncytial layer, ensuring proper placental morphogenesis and maternal–fetal interface establishment [61]. The coordinated activity of these angiogenic networks ensures proper uteroplacental perfusion, villous maturation, and the structural foundation necessary for optimal fetal growth. Disruption of this finely tuned angiogenic–trophoblastic axis has been implicated in placental insufficiency disorders such as preeclampsia and intrauterine growth restriction [61].

In the placenta, VEGF-A influences the proliferation and differentiation of trophoblast, regulates the stability of nascent capillaries, and promotes endothelial proliferation and vascular permeability [53]. From a molecular perspective, members of the VEGF family interact with one or more receptor tyrosine kinases as well as with specific co-receptors. Almost all known cellular effects of VEGF-A are mediated by vascular endothelial growth factor receptor 2 (VEGF-R2). The function of vascular endothelial growth factor receptor 1 (VEGFR1) is less well understood, but it likely acts by modifying VEGFR2 function, while also serving as a “pseudoreceptor”, preventing the binding of VEGF to VEGFR2. VEGFR1 can also form heterodimers with VEGFR2, further modulating its activity and the downstream signaling outcome [25]. The interaction of anti-angiogenic VEGF isoforms with VEGFR1 and VEGFR2 thus represents a finely tuned mechanism for balancing vascular proliferation and quiescence. Through this regulatory interplay, VEGF splicing serves as a key post-transcriptional control point that determines the angiogenic phenotype under both physiological and pathological conditions [25].

PlGF is a member of the cysteine-knot growth factor family and exerts both proinflammatory and angiogenic roles [62]. In early pregnancy, it is important for placental angiogenesis, as well as for the induction, differentiation and invasive migration of trophoblasts into the maternal decidua. PlGF promotes angiogenesis, increases under ischemic conditions, and activates inflammatory cells. PlGF is synthesized in the placenta and enhances the activity of VEGF-A [63]. Maternal PlGF concentrations increase significantly at the beginning of pregnancy, peak in the middle of the pregnancy and then gradually decrease towards term [64,65,66,67]. In women who are going to develop PE, this decrease in the concentration of PlGF occurs prematurely, usually before the onset of symptoms. There is a strong correlation between PlGF levels and the tension of oxygen; thus, in placental hypoxia, the levels of PlGF are decreased accounting for the reduced levels observed in women with PE [64,65].

An extremely important receptor is sFlt-1 (the soluble form of VEGF-R1), which has an essential role in placental angiogenesis during early pregnancy. It activates the development, differentiation and invasion of trophoblast into the maternal decidua [68]. sFlt-1 binds VEGF and PlGF, reducing their circulating levels and inhibiting their action, thereby causing endothelial dysfunction. The placenta is the main source of the sFLt-1 in pregnant women [69]. In normal pregnancy, sFlt-1 concentrations increase steadily during the third trimester; however, in women who will develop PE, they increase prematurely. High concentrations of sFlt-1 have been found in pregnant women with PE before the appearance of other manifestations, such as hypertension or proteinuria [66]. The increase in concentration of maternal sFlt-1 occurs roughly five weeks prior to the appearance of symptoms and can reach levels up to five times higher than in normal pregnancy [68].

In normal pregnancies, reference values for these biomarkers have been established in serum. In women with PE, altered levels of these biomarkers have been found compared to normal pregnancies, suggesting their participation in the pathophysiology of preeclampsia [41,64].

## 4. Role of sFlt-1/PlGf Ratio in Preeclampsia

The multifactorial etiology of PE highlights the importance of identifying clinically relevant biomarkers for the diagnosis and prognosis of the disease [67]. Over the last 20 years, many biomarkers based on the fundamental pathophysiological mechanism of PE have been proposed to predict preeclampsia in high-risk women [70,71]. The concentration of these factors in maternal blood may be increased or decreased early in pregnancy, before the clinical symptoms of preeclampsia develop. However, the literature on these biomarkers is not yet sufficiently reliable for use in regular clinical settings [72]. Moreover, during pregnancy, plasma VEGF levels are low and usually below detectable limits by most commercial detection kits [70,71].

Ιn women with PE, many studies have observed elevated levels of sFlt-1 and reduced levels of PlGF in the maternal serum during early pregnancy, indicating a blockade of PlGF action by sFlt-1 [73,74,75]. Recently, the sFlt-1/PlGf ratio has been introduced as a screening biomarker that is regarded as having a very promising role in the prognosis and management of PE [76,77,78,79,80,81,82,83,84,85,86,87,88,89,90,91,92] (Table 3). According to studies, the diagnostic value of this ratio is significantly greater than that of each biomarker separately [76]. Although VEGFA and sEng, both angiogenic biomarkers, have been investigated as predictive and diagnostic biomarkers, they have limited clinical usefulness as they do not provide additional clinical value [66,93].

In 2003, attention was drawn to the sFlt-1/PlGf ratio for the first time when it was published by Maynard et al. that women with PE had five times higher sFlt-1 levels compared to normal pregnancies. Furthermore, sflt-1 levels decrease 48 h after delivery, suggesting that it is produced by the placenta. In addition, in PE patients, decreased levels of free serum VEGF and PlGF were found compared to normal pregnancies, and this reduction was in proportion to the increased levels of serum sFlt-1 [41].

In 2004 Levine et al. observed that the levels of sflt-1 were elevated five weeks prior to the onset of PE, while at the same time they noted a decrease in the levels of PlGF and VEGF concluding that there is a correlation between these biomarkers and both the prediction and severity of PE [64].

Ιn 2005, Chaiworapongsa et al. suggested that sFlt-1 measurement is most informative approximately one month prior to clinical diagnosis [73], whereas Thadhani et al. indicated that the optimal timing for measurement is between 28 and 32 weeks for early- onset and 30–34 weeks for late-onset PE [94].

Moreover, in 2005, Hirashima et al. determined reference values for sFlt-1, free PlGF, and the sFlt-1/PlGF ratio using a commercially available ELISA kit. Reference curves were constructed for the entire duration of pregnancy, applying a 90% confidence interval. According to their findings, in normal pregnancy, free PlGF levels increase early (8–12 weeks) and decrease towards the end of pregnancy (35–39 weeks). In contrast, sFlt-1 levels decrease initially (8–20 weeks), then gradually increase (26–30 weeks), peaking at 35–39 weeks [56]. The establishment of the sFlt-1/PLGF ratio contributed to the rapid automation of tests that utilize this indicator to improve the prognosis and diagnosis of PE. All initial studies were based on ELISA kits and were not directly applicable to clinical settings. The demand for rapid and accurate diagnosis led to the development of automated assays for measuring sFlt-1 and PlGF.

In 2010, Ohkuchi et al., employing an automated electrochemiluminescence immunoassay system, found that the sFlt-1/PlGF ratio provides significant diagnostic value for both early- and late-onset PE, with an optimal cutoff value of 85 for late-onset PE [74]. Subsequently, evaluations of the new automated Elecsys method (Roche Diagnostics, GmbH) reported a sensitivity of 82% and a specificity of 95% for the 85 cutoff, while for early-onset PE, the sensitivity was 89% and the specificity 97% [95]. A later study employing a new automated immunoassay system (Beckman Coulter), confirmed these findings [96].

The first clinical trial to use Elecsys immunoassays for the determination of sFlt-1 and PlGF in order to evaluate the sFlt-1/PlGF ratio for the prediction of PE in short-term (up to 4 weeks) was the PROGNOSIS (Prediction of short-term Outcome in Pregnant Women with Suspected Preeclampsia) study. It was carried out in 14 countries from 2010 to 2013 and reported that in pregnant women < 37 weeks of gestation, an sFlt-1/PlGF ratio ≤ 38 reliably excludes the occurrence of PE in the following week, demonstrating a negative predictive value (NPV) of 99.3%, sensitivity of 80%, and specificity of 78.3%. Moreover, a ratio > 38 predicts the occurrence of PE or HELLP syndrome within 4 weeks, with a positive predictive value of 36.7%, sensitivity of 66.2%, and specificity of 83% [87].

A post hoc analysis of PROGNOSIS data showed that an sFlt-1/PlGF ratio ≤ 38 excluded the occurrence of PE for up to 4 weeks in women with suspected PE (24+ 0–36 + 6 weeks) with an NPV of 94.3%. Repeated measurements can further assess the risk [88]. PROGNOSIS Asia, carried out in 25 Asian centers, confirmed that an sFlt-1/PlGF ≤ 38 excludes PE within 1 week (NPV 98.6% sensitivity 76.5%, specificity 82.1%), while a ratio > 38 is predictive of PE over the next 4 weeks (PPV 30.3%, sensitivity 62%, specificity 83.9%) [89].

In 2021 Droge et al. used the ratio sFlt-1/PlGF as a “traffic light’ system to predict preeclampsia and guide clinical management. Women with a ratio below 38 (green light) have a low risk of developing preeclampsia in the next 4 weeks, and follow-up is recommended within that period. Those with a ratio between 38 and 85 (yellow light) have a moderate risk, and retesting after one week is advised. Women with a ratio above 85 (red light) are at high risk and should undergo immediate hospital evaluation, with possible admission depending on ultrasound, cardiotocography and laboratory results [62]. This ratio is particularly useful between 24 and 34 weeks of gestation, allowing for closer monitoring and more informed decisions regarding further diagnostics and treatment [85,97].

The INSPIRE study was the first randomized clinical trial to evaluate the sFlt-1/PlGF ratio with a cutoff of 38 for the short-term prediction of PE. This ratio showed a sensitivity and NPV of 100%, compared to 83.3% and 97.8%, respectively, when clinical assessment alone was applied. These results confirm that combining the sFlt-1/PlGF ratio with standard clinical evaluation allows the accurate identification of high-risk women without increasing the number of hospital admissions [90]. Moreover, in a post hoc analysis of the INSPIRE trial, a cutoff 85 of the sFlt-1/PlGF ratio was shown to predict the onset of PE within 4 weeks with a PPV of 71.4%. These findings suggest that applying the ≥85 cutoff for prediction together with the 38 cutoff for exclusion could enhance the clinical management of women with suspected PE [98].

In a secondary analysis of the INSPIRE study, Kifle et al. developed a logistic regression model to predict the onset of PE using sFlt-1, PlGF, and the sFlt-1/PlGF as continuous variable, as well as the sFlt-1/PlGF ratio categorized by the 38 cutoff. They reported that models based on the continuous values of sFlt-1 or the sFlt-1/PlGF ratio demonstrated superior predictive accuracy compared with those using PlGF alone or the sFlt-1/PlGF ratio categorized at 38 [91].

**Figure 2:**
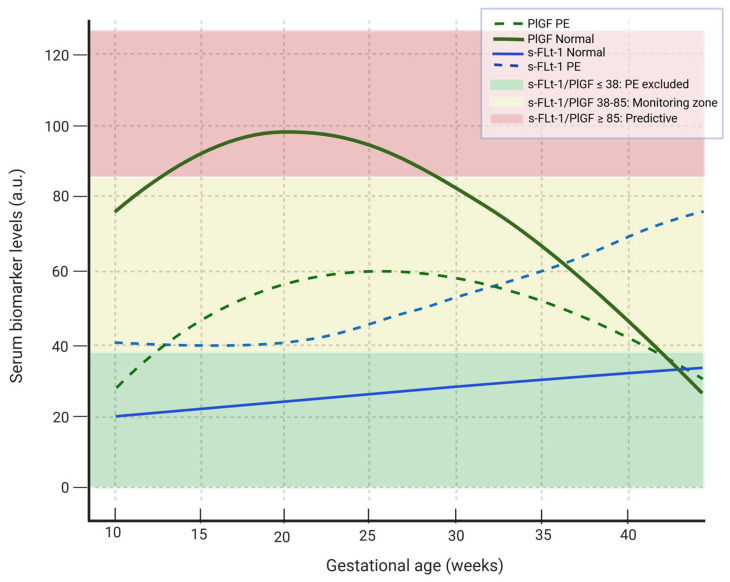
Maternal serum dynamics of sFlt-1 and PlGF in normal pregnancy and preeclampsia. In women with PE, the PlGF shows a smaller and later increase while the sflt-1 rises earlier and in larger quantities compared with normal pregnancies. Green fond: sFlt-1/PlGF ratio ≤ 38 --> PE excluded, yellow fond: sFlt-1/PlGF ratio 38–85 --> moderate risk for PE, red fond: sFlt-1/PlGF ≥ 85 --> predictive of PE. The continuous green line shows the variability of PlGF levels in normal pregnancy, the dashed green line indicates the variability of PlGF levels in PE, the solid blue line indicates the variability of sFlt-1 levels during normal pregnancy, the dashed blue line indicates the variability of sFlt-1 level in PE pregnancy. PlGF: platelet growth factor, sFlt-1: soluble fms-like tyrosine kinase-1. (Created with Biorender.com).

Huges et al. tried to determine if an Flt-1/PlGF ratio threshold of 38, measured at <37 + 0 weeks of gestation, could reliably exclude PE within 1 week, predict its onset within 4 weeks and provide prognostic information on prenatal outcomes. They reported that among women <37 weeks, an sFlt-1/PlGF ratio ≤ 38 ruled out preeclampsia the following 1 week (NPV 96.2%) and predicted disease over the following 4 weeks (PPV 75%) highlighting its clinical value and the need for adoption in national protocols [92] (Figure 2).

### Placental Biomarkers in the Prediction and Pathogenesis of Preeclampsia

Additional placental proteins have emerged as promising candidates. Annexin A2 (ANXA2), a calcium-dependent phospholipid-binding protein, contributes to trophoblast invasion and spiral artery remodeling, both essential for adequate placental perfusion [99]. Reduced ANXA2 expression impairs these processes, leading to placental hypoperfusion and adverse outcomes such as fetal growth restriction, preterm birth, and PE [100]. Specifically, Ya-Nuan Chen remarked that ANXA2 levels below 6.13 ng/mL were associated with earlier deliveries (median 32 weeks), while higher levels corresponded to longer gestation. Combining ANXA2 with the sFlt-1/PlGF ratio improved risk stratification, showing higher rates of preterm and cesarean deliveries in the high-risk group and demonstrating strong predictive value for adverse outcomes (AUC = 0.858), outperforming the sFlt-1/PlGF ratio alone [101]. Similarly, Growth Differentiation Factor 15 (GDF-15), a member of the TGF-β superfamily, is highly expressed in the placenta and upregulated under hypoxic and inflammatory conditions [102]. Elevated circulating GDF-15 levels have been observed in women with preterm PE. Data from the Fetal Longitudinal Assessment of Growth (FLAG) study demonstrated that combining GDF-15 with the sFlt-1/PlGF ratio significantly improved predictive performance (sensitivity 68.3%, specificity 83.2%) compared to either marker alone [103].

Moreover, placental-derived proteins play a central role in mediating implantation, vascular remodeling, and maternal–fetal communication, and their dysregulation contributes significantly to the development of preeclampsia (PE). Among the most extensively investigated is Placental Protein 13 (PP13), synthesized by the syncytiotrophoblast, which supports trophoblast invasion and modulates maternal immune tolerance during early placentation [104,105]. Experimental data suggest that PP13 induces apoptosis in activated T cells and macrophages within the decidua, facilitating local immune adaptation. Several clinical studies have demonstrated that first-trimester PP13 levels are lower in women who later develop PE compared with normotensive pregnancies [106]. A pooled analysis of Vasilache et al. showed a sensitivity of 0.53 and a specificity of 0.83 for PP-13 in predicting PE [107]. Meta-analytic data confirm its predictive potential, particularly for early-onset forms of the disease, underscoring its utility in early risk stratification [108].

Another important biomarker, Pregnancy-Associated Plasma Protein A (PAPP-A), is a protease that cleaves insulin-like growth factor-binding proteins, thereby increasing the bioavailability of insulin-like growth factors (IGFs), which are essential for placental and fetal growth [109]. Low first-trimester PAPP-A concentrations have been consistently linked to small-for-gestational-age infants, spontaneous preterm birth, and PE [110,111]. A systematic review and meta-analysis by Tzanaki et al. highlighted the strong potential of PAPP-A as an early biomarker for PE, facilitating the timely identification of high-risk women and enabling preventive strategies [112]. Although PAPP-A alone lacks strong discriminatory power, combining it with other biomarkers enhances predictive performance and supports individualized monitoring during pregnancy [113]. Moreover, Hughes et al. reported that a first-trimester combining PAPP-A, PlGF, sFlt-1, and clinical risk factors achieved high accuracy for preeclampsia screening, though cost and complex ultrasound limit its use [114].

Alpha-fetoprotein (AFP), primarily synthesized by the fetal liver, has also been implicated in placental dysfunction. Elevated maternal serum AFP levels during the second trimester are believed to reflect increased placental permeability or structural compromise rather than direct fetal effects [115]. The combination of AFP and PAPP-A has shown improved predictive ability, as a high AFP/PAPP-A ratio is associated with an increased risk of severe PE, though overall performance remains modest [116]. Moreover, the predictive efficiency of the combined detection of AFP, PLGF, b-hCG and PAPP-A was significantly increased. These findings emphasize that no single marker can capture the multifactorial nature of PE, reinforcing the importance of composite biomarker panels [115].

Collectively, these findings indicate that integrating multiple placental protein biomarkers—such as PP13, PAPP-A, AFP, ANXA2, and GDF-15—with angiogenic factors like sFlt-1 and PlGF could substantially improve the early prediction and risk stratification of preeclampsia.

Moreover, Sclerostin, encoded by the SOST gene, is produced by osteocytes and chondrocytes and inhibits bone formation by locking the Wnt signaling pathway [117,118]. Godang et al. reported than umbilical cord sclerostin levels were higher than maternal levels and significantly predicted neonatal bone mineral content [119].

## 5. First-Trimester Screening for PE

The main goal of first-trimester screening is to identify women at increased risk of developing PE, enabling the timely implementation of appropriate preventive interventions. However, in numerous clinical centers, the routine use of such screening protocols remains limited, and risk assessment is often based solely on clinical factors, according to the guidelines of ACOG 2018 and NICE 2019 [11,12]. Extensive work has focused on creating first-trimester prediction models for PE, which still require evaluation and external validation across diverse populations [14].

According to the International Federation of Gynecology and Obstetrics (FIGO) guidelines, all pregnant women should be screened for PE in the first trimester through the assessment of clinical risk factors and maternal biomarkers [120]. The Fetal Medicine Foundation (FMF) has developed one of the most well-documented algorithms for the first trimester prediction of PE, which has been validated in two large multicenter trials [121,122]. This algorithm is based on the combination of clinical risk factors, maternal age, mean arterial pressure (MAP), mean uterine artery pulsatility index (mean UtA-PI) measurements, and maternal PIGF. Although it is primarily applied in the first trimester, it can be adapted for risk assessment at subsequent stages of pregnancy [123,124,125,126].

Sflt-1 is not considered a reliable marker for the first-trimester screening of preeclampsia, since its levels typically become elevated only after 21–24 weeks of gestation in women who will eventually manifest the condition [64].

Beyond protein-based molecules, RNA-derived biomarkers have emerged as critical regulators and potential diagnostic tools in the context of PE.

Circular RNAs (circRNAs) are covalently closed, single-stranded RNA molecules that act as sponges for specific microRNAs (miRNAs), thereby influencing downstream gene expression. Recent studies have identified circRNA_06354 as a critical regulator of early-onset PE (EOPE). This circRNA participates in the circRNA_06354/hsa-miR-92a-3p/VEGF-A signaling pathway, modulating vascular endothelial growth factor A (VEGF-A) expression. Dysregulation of this axis impairs trophoblast invasion and migration into the spiral arteries, leading to defective placentation under hypoxic conditions. Consequently, circRNA_06354 has been proposed as a novel pathogenic and predictive biomarker for EOPE [127].

MicroRNAs (miRNAs), particularly those within the chromosome 19 microRNA cluster (C19MC)—one of the largest miRNA clusters in the human genome—play pivotal roles in placental development. These placenta-specific miRNAs regulate trophoblast differentiation, invasion, and angiogenesis, with expression levels increasing throughout gestation [128,129]. The dysregulation of specific C19MC members contributes to PE pathogenesis: miR-519d suppresses trophoblast invasion and migration, miR-515-5p inhibits syncytiotrophoblast differentiation, miR-517a/b/c and miR-517-5p reduce trophoblast invasiveness and enhance sFlt-1 secretion, while miR-518b promotes trophoblast proliferation [130]. Additionally, miR-520h has been associated with gestational hypertension-related PE [131]. Aberrant exosomal miRNA profiles can disrupt maternal immune tolerance and promote systemic inflammation, further contributing to endothelial dysfunction. Circulating C19MC miRNAs, therefore, hold substantial promise as predictive and diagnostic biomarkers of placental dysfunction and PE [132].

Long non-coding RNAs (lncRNAs) have also gained attention as key epigenetic regulators in PE. These molecules influence gene transcription, RNA stability, and chromatin organization, thereby modulating trophoblast behavior and placental function [133]. Distinct lncRNA profiles appear to characterize early- versus late-onset PE, suggesting different molecular mechanisms underlying these clinical subtypes. Although several circulating lncRNAs—such as BC030099, AF085938, G36948, and AK002210—have been proposed as biomarkers, their low abundance and the absence of standardized detection methods currently limit their clinical application [132].

## 6. Clinical and Economic Impact of sFlt-1/PlGf Ratio Testing: Pharmacoeconomic Justification

Several international guidelines recommend the use of the maternal sFlt-1/PlGF ratio as a tool for both the diagnosis and prediction of PE. These include the NICE 2016 guidelines [134], the European Society of Cardiology (ESC) 2018 guidelines [135] as well as national Societies such us the Danish Society for Obstetrics and Gynecology 2018 [136], the Swiss Society for Obstetrics and Gynecology (SGGG) 2019, the Austrian Society of Obstetrics and Gynecology (OeGGG) 2019, the German Society of Obstetrics and Gynecology (DGGG) [137] and the Spanish Society of Gynecology and Obstetrics (SEGO) 2020 guidelines [138].

Beyond their clinical value, the implementation of the sFlt-1/PlGF ratio test has demonstrated substantial economic benefits across multiple healthcare systems. Zeisler et al. reported that thesFlt-1/PlGF ratio could serve as a valuable diagnostic indicator for PE, helping to reduce costs, shorten hospital stays, avoid unnecessary interventions and preterm births, and ensure that care is directed to patients genuinely at risk [79].

Building on these findings, several country-specific evaluations have further quantified the economic advantages of introducing the test into routine practice. In the UK, it is estimated that the test could save approximately USD 344 per patient by reducing unnecessary hospital admissions among women at low risk of PE, as a result of improved diagnostic accuracy, while enabling timely identification and the appropriate management of higher risk cases [139]. Similarly, an economic model in Brazil, evaluating both public and private healthcare perspectives, showed cost savings of BRL 185.06–BRL 635.84 per patient compared with current diagnostic approaches, primarily due to improved diagnostic accuracy and fewer unnecessary hospitalizations [140]. In Belgium, the introduction of the test in the public healthcare system is expected to save EUR 172 per patient by avoiding admissions of women with suspected PE who do not progress to the condition [141]. Under the German DRG system, use of the sFlt-1/PlGF ratio test (cutoff 38) could reduce hospitalizations from 44.6% to 24%, generating EUR 361 per patient in savings [142].

Across all settings, the test supports better clinical decision-making, optimizes resource use, and ensures that high-risk women receive appropriate management while low-risk women avoid unnecessary hospitalization. Moreover, resource allocation is improved, as prioritization of high-risk pregnancies allows for more efficient use of intensive care units’ capacity.

## 7. Conclusions

The recognition and management of women at increased risk of PE is an evolving field. Current research aims to predict the onset of PE, particularly severe cases, at an early stage, enabling timely intervention and reducing maternal morbidity and mortality.

The sFlt-1/PlGF ratio has emerged as a valuable biomarker for both the diagnosis and prediction of PE. It is a rapid, reliable and cost-effective tool that can be readily implemented in clinical practice. Its use has demonstrated clear pharmacoeconomic benefits by improving diagnostic accuracy, supporting risk stratification, enabling targeted interventions and reducing unnecessary hospitalizations. This contributes to a more efficient utilization of healthcare resources in the management of suspected PE.

Incorporation of the sFlt-1/PlGF ratio test into the care of high-risk pregnancies could reduce maternal and neonatal complications, improve clinical decision-making, and generate substantial cost savings. Moreover, it decreases anxiety and medical interventions in low-risk patients, while the early detection of high-risk patients facilitates timely transfer to tertiary care centers.

At the health system level, benefits include shorter hospital stays for both mothers and infants, fewer unnecessary diagnostic tests, imaging, and monitoring in cases of false positive results, and more efficient monitoring strategies. This approach not only improves patient outcomes but also supports value-based care models and helps payers control costs. For maximum effectiveness, adoption of the sFlt-1/PlGF ratio should be guided by national guidelines and diagnostic algorithms, ensuring consistent and appropriate clinical use.

## 8. Future Directions

The sFlt-1/PlGF ratio has been widely acknowledged as a valuable biomarker for the early detection and prognosis of preeclampsia (PE); nonetheless, several critical research gaps persist. Evidence indicates inconsistent diagnostic thresholds among populations, influenced by gestational age, ethnicity, and assay techniques. The predictive performance for late-onset and mild PE remains uncertain, as most studies primarily address severe, early-onset cases. Moreover, long-term and integrative investigations that track serial biomarker changes alongside imaging or clinical parameters are still limited. A noticeable lack of studies in low-resource settings further restricts the global applicability of current findings. The prognostic role of the ratio after diagnosis, particularly in predicting disease progression or maternal recovery, is not yet clearly defined. In addition, many analyses exclude women with coexisting conditions such as diabetes, renal disease, or multiple pregnancies, which undermines external validity. Economic analyses and long-term cardiovascular follow-up studies are also insufficient [143,144,145,146]. Collectively, these issues emphasize the necessity for standardized, multicenter, and longitudinal research frameworks that combine biomarker, imaging, and clinical data to improve both diagnostic accuracy and prognostic reliability. To achieve more precise prediction, diagnosis, and management of PE, deeper insights into the pathophysiological pathways and angiogenic mechanisms are essential. Furthermore, large-scale genomic and proteomic investigations are needed to identify and validate new biomarkers suitable for clinical implementation. For effective PE prevention, the routine use of the sFlt-1/PlGF ratio in early screening protocols should be integrated into national healthcare systems worldwide.

## Figures and Tables

**Table 1:** Risk factors for preeclampsia. Maternal risk factors for preeclampsia according to professional organizations ACOG 2018, NICE 2019, ISSHP 2018 and [14,15,16]. BMI: body mass index, FGR: fetal growth restriction.

High Risk Factor for PE	Moderate Risk Factors for PE
Prior preeclampsia	First pregnancy
Chronic hypertension	Maternal age > 40 years
Pregestational diabetes mellitus	Multifetal pregnancy
Chronic kidney disease (including kidney-transplanted women)	Prior pregnancy with placental abruption
Antiphospholipid syndrome/Systemic lupus erythematosus	Prior pregnancy with stillbirth
Multiple gestation	Prior pregnancy with FGR
Pre-pregnancy BMI > 30	Genetic susceptibility
	Assisted reproductive technology
	Family history of thrombophilia
	Pregnancy interval of >10 years
	Family history of preeclampsia
	Trisomy 13 fetus
	Maternal low birth weight Race

**Table 2:** Role of endothelial dysfunction in the pathogenesis of PE.

Author	Ref.	Role of Endothelial Dysfunction in Pathogenesis of PE
Jim B. et al., 2017	[17]	Central role in PE pathogenesis; caused by antiangiogenic imbalance (↑ sFlt-1, ↑ sEng → ↓ VEGF/PlGF); leads to hypertension, proteinuria, and long-term vascular risk.
Stepan H. et al., 2023	[18]	Endothelial dysfunction due to angiogenic imbalance (↑ sFlt-1/↓ PlGF) causes vasoconstriction, increased permeability, and organ injury in PE.
Redman C.W. et al., 2005	[19]	Systemic endothelial activation and injury—central to maternal syndrome; causes vasoconstriction, hypertension, and proteinuria in PE.
Palei A.C. et al., 2013	[20]	Placental ischemia–induced endothelial dysfunction (↓ NO, ↑ endothelin-1, ↑ sFlt-1) drives vasoconstriction and hypertension in PE.
Brosens I. et al., 1967	[21]	Defective spiral artery remodeling → placental ischemia → endothelial dysfunction underlying PE.
Brosens I. et al., 2011	[22]	Abnormal deep placentation → placental ischemia → endothelial dysfunction → PE and other obstetrical syndromes.
Taylor R.N. et al., 2003	[23]	Low PlGF → impaired placental angiogenesis → endothelial dysfunction → PE.
Taylor R.N. et al., 2009	[24]	VEGF promotes endothelial proliferation, angiogenesis, and tissue repair.
Ntellas P. et al., 2020	[25]	VEGF/VEGFR overactivation → Endothelial dysfunction → ↑ vascular permeability, inflammation, hypoxia → tumor progression, metastasis.
Koch S. et al., 2012	[26]	Endothelial dysfunction from abnormal VEGR signaling → ↑ permeability and disrupts vascular integrity.
Boudria A. et al., 2019	[27]	Endothelial dysfunction induced by the VEGF165b/β1 integrin-VEGFR autocrine loop enhances tumor progression and resistance to anti-angiogenic therapy.
Woolard J. et al., 2009	[28]	Altered VEGF-A isoform balance → endothelial dysfunction vascular permeability, stability, and signaling.
Zhou Y. et al., 1997	[29]	Impaired cytotrophoblast vascular adhesion and invasion → endothelial dysfunction in PE → defect vascular remodeling.
Brosens I. et al., 1972	[30]	Endothelial dysfunction → placental infracts in PE->inadequate spiral artery remodeling and ↓ placental perfusion.
De Wolf F. et al., 1975	[31]	Endothelial dysfunction in hypertensive pregnancies → acute atherosis impairing spiral arteries and placental flow.
Huang Q.T. et al., 2013	[32]	Oxidative stress → endothelial dysfunction → ↑ sFlt-1 expression in trophoblast → PE.
Vaughan J.E. et al., 2002	[33]	Oxidative stress → endothelial dysfunction, → vasoconstriction, ↑ vascular permeability, and inflammation.
Romero R. et al., 2013	[34]	Endothelial dysfunction → ↓ bioavailability of vasodilators (e.g., NO, prostacyclin) and ↑ vasoconstrictors (e.g., endothelin-1), → hypertension, ↑ vascular permeability and organ injury.
Rajakumar A. et al., 2004	[35]	HIFs are overexpressed and functionally active in preeclamptic placenta, contributing to altered gene expression under low oxygen conditions.
Caniggia I. et al., 2000	[36]	Sustained HIF-1α/TGF-β3 signaling impairs trophoblast invasion and spiral artery remodeling, → to abnormal placental perfusion → endothelial dysfunction, hypertension and organ injury.
Caniggia I. et al., 2019	[37]	Abnormal placental factors (e.g., excess sFlt-1) → endothelial dysfunction, → vasoconstriction, ↑ permeability, and organ injury.
Caniggia I. et al., 2018	[38]	Imbalance between angiogenic factors (e.g., placental growth factor) and anti-angiogenic factors (e.g., soluble endoglin, soluble VEGF receptor-1) → endothelial dysfunction → impairs vascular remodeling and ↑ vascular resistance → hypertension and organ injury.
Powe C.E. et al., 2011	[39]	Imbalance between pro-angiogenic and anti-angiogenic factors →, maternal endothelial dysfunction → hypertension, proteinuria, and ↑ cardiovascular risk later in life.
Ahmad S. et al., 2004	[40]	↑ levels sFlt-1 in preeclamptic placental tissue inhibit angiogenesis by sequestering VEGF and placental growth factor (PlGF), → impaired endothelial cell migration and tube formation.
Maynard S.E. et al., 2003	[41]	↑ sFlt1 → endothelial dysfunction by sequestering vascular endothelial growth factor (VEGF) and placental growth factor (PlGF), → hypertension, proteinuria, and glomerular endotheliosis.
Walsh S.W. et al., 2004	[42]	Imbalance between TXA_2_ and (PGI_2_), favoring TXA_2_ → hypertension, platelet aggregation, and ↓ uteroplacental blood flow. Oxidative stress exacerbates this imbalance by ↑ TXA_2_ synthesis and ↓ PGI_2_ synthesis. Low-dose aspirin therapy has been considered for the prevention of preeclampsia because it selectively inhibits TXA_2_ synthesis.
Shah D. et al., 2015	[43]	Placental ischemia → release of bioactive factors into the systemic circulation → widespread endothelial dysfunction. This dysfunction is characterized by ↓ NO and PG levels, ↑ ET-1 and TX A_2_ levels, and ↑ vascular smooth muscle contraction, culminating in hypertension and organ damage.
Su H. et al., 2024	[44]	ET-1 induces stronger contractions in placental veins through ETAR/ETBR receptors, despite ↓ CaV1.2 expression. Hypoxia ↑ ET-1 levels, promoting caldesmon (CALD1) expression and ↑ vascular tone → placental vascular dysfunction.
Zolfaghari M.A. et al., 2021	[45]	An imbalance between Th17 and Treg cells, regulated by microRNAs → endothelial dysfunction and systemic inflammation. ↑ Th17 and ↓ Treg cell numbers, along with ↑ IL-17 and ↓ IL-10 levels, are observed in preeclamptic patients. Additionally, the upregulation of miR-106b and miR-326 has been linked to this imbalance.
Aneman I. et al., 2020	[46]	EOPE is associated with impaired placental development and growth restriction, while LOPE is linked to maternal endothelial dysfunction
Regal J.F. et al., 2017	[47]	In PE, deregulated complement activation → endothelial dysfunction, hypertension, and placental insufficiency. C5 inhibition → potential therapeutic approach.
Phipps E.A. et al., 2019	[48]	sFLT-1and sENG released into maternal circulation disrupt VEGF/PlGF signaling, lowering production of vasodilators (e.g., nitric oxide, prostacyclin) → vasoconstriction, proteinuria, hypertension and multi-organ injury.
Youssef L. et al., 2021	[49]	↑ activation of complement and coagulation cascades → deposition of C5b-9 and vWF on endothelial cells exacerbating endothelial injury and dysfunction in early onset severe PE.
Alpoim P.N. et al., 2011	[50]	↑ vWF levels and ↓ ADAMTS13 activity → microvascular thrombosis and endothelial injury.
Venou T.M. et al., 2024	[51]	↓ ADAMTS13 activity and elevated complement activation (C5b-9)**,** → microvascular thrombosis, complement-mediated endothelial damage, and vascular injury in preeclampsia/HELLP syndrome

↑: increased, ↓: decreased, sFlt-1: soluble fms-like tyrosine kinase-1, VEGF: vascular endothelial growth factor, PlGF: platelet growth factor, PE: preeclampsia, NO: nitic oxide, VEGFR: vascular endothelial growth factor receptor, HIFs: hypoxia-inducible transcription factors, HIF-1α/TGF-β3: hypoxia-inducible transcription factor-1α/transforming growth factor beta-3, TXA_2_: thromboxane A2, PGI_2_: prostaglandin A2, ET-1: endothelin-1, ETAR/ETBR: endothelin A type receptor/endothelin B type receptor, CaV1.2: voltage-dependent L-type calcium channel subunit alpha—1C, Th17: T helper 17 cells, IL-17: interleukin—17, IL-10: interleukine-10, miR-106b: microRNA-106b, miR-326: microRNA-326, EOPE: early onset of preeclampsia, LOPE: late onset of preeclampsia, C5: complement C5, sENG: soluble endoglin, C5b-9: complement complex C5b-9, vWF: von Willebrand factor.

**Table 3:** sFlt-1/PlGF ratio as a diagnostic biomarker for the prediction of PE.

Author, Year	Ref.	GA/Sample Size	Mean BP (mmHg)	Findings—Key Conclusions
Verlohren et al., 2014	[77]	10–37 w 1149 women	---------	Developed gestational age-specific cutoffs; ratio ≥ 85 (<34 w) and ≥110 (≥34 w) linked to PE. Gestational age-specific cutoffs improve diagnostic accuracy.
Álvarez-Fernández et al., 2014	[78]	<34 w (early-onset PE focus) 257 women	Mild PE: SBP: 145 DBP: 91 Severe PE: SBP: 167 DBP: 106	Identified angiogenic factors as biomarkers for early PE and adverse pregnancy outcomes. sFlt-1/PlGF ratio improves early diagnosis and predicts imminent delivery.
Hund et al., 2014	[87]	24–37 w without clinical suspicions. 1000 women	---------	Adoption of the sFlt-1/PlGF test in clinical practice has the potential to reduce the frequency of adverse pregnancy outcomes for both mother and fetus, and decrease healthcare costs associated with the unnecessary hospitalization of women with suspected preeclampsia.
Zeisler et al., 2016	[79]	24 + 0–36 + 6 w 1050 women	---------	sFlt-1/PlGF ratio ≤ 38 ruled out PE within 1-week (NPV 99.3%). Ratio is reliable for short-term prediction, helps exclude PE.
Zeisler et al., 2019	[88]	24 + 0–36 + 6 w 550 women	Development Cohort: NO PE: SBP: 128 DBP: 80 PE: SBP: 137 DBP: 85 Validation cohort: NO PE: SBP: 125 DBP: 78 PE: SBP: 137 DBP: 90	A sFlt-1/PlGF ratio ≤ 38 effectively excluded the onset of PE for up to 2 and 3 weeks after baseline (NPVs of 97.9% and 95.7%, respectively). Within 4 weeks, the development of pre-PE was also reliably ruled out, NPV 94.3%.
Bian et al., 2019	[89]	20 + 0 (18 + 0 in Japan)–36 + 6 w 764 women	NO PE: SBP: 132 DBP: 81 PE: SBP: 144 DBP: 90	An sFlt-1/PlGF ratio of ≤38 had an NPV of 98.9% (95% CI, 97.6–99.6%) for ruling out fetal adverse outcomes within 1 week and a ratio of >38 had a positive predictive value of 53.5% (95% CI, 45.0–61.8%) for ruling in fetal adverse outcomes within 4 weeks. The sFlt-1/PlGF ratio cutoff of 38 demonstrated clinical value for the short-term prediction of preeclampsia in Asian women with suspected preeclampsia, potentially helping to prevent unnecessary hospitalization and intervention.
Cerdeira et al., 2019	[90]	24 + 0–37 + 0 w 370 women with suspected PE	Non-reveal arm: SBP: 132 DBP: 180 Reveal arm: SBP: 131 DBP: 84	The use of the sFlt-1/PlGF ratio enhanced clinical accuracy while leaving admission rates unchanged.
Perry et al., 2020	[80]	>20 w 302 pregnant women with hypertension	Not Delivered within 4 days: 107 Delivery within 7 days: 111.5 Delivery within 14 days: 110.8	sFlt-1/PIGF ratio combined with the maternal characteristics predicts delivery within 1 or 2 weeks in GA < 35 weeks. Angiogenic biomarkers add predictive value, especially when the sFlt-1/PlGF ratio is applied as a continuous measure, though their usefulness declines after 35 weeks of gestation.
Andersen et al., 2021	[81]	≥20 wk 517 women	---------	Kryptor sFlt-1/PlGF thresholds validated in real-life. Ratio cutoffs applicable in clinical routine. The sFlt-1/PlGF ratio is a useful clinical tool for ruling out and ruling in preeclampsia within 1 week.
Soundararajan et al., 2021	[82]	28–37 w 50 women	sFlt-1/plGf > 85: SBP: 157 DBP: 100	Pregnancy management guided by these biomarkers enabled the closer monitoring of women at highest risk for adverse outcomes, while allowing standard follow-up for those at lower risk.
Peguero et al., 2021	[83]	<34 w 63 women	------------	Longitudinal changes in sflt-1 added prognostic value. Serial measurements of sflt-1 improve prediction of adverse outcomes.
Drögeetal. et al., 2021	[84]	20–37 w 1117 women	No AO: SBP: 127.4 DBP: 79.6 AO: SBP: 138.3 DBP: 86.4	Risk-stratification with the sFlt-1/PlGF cutoff values into high- (>85), intermediate- (38–85), and low-risk (<38) showed a significantly shorter time to delivery in high- and intermediate- versus low-risk patients. Ratio predicted PE-related adverse outcomes in real-world setting. High clinical utility for predicting maternal/fetal complications.
Jeon et al., 2021	[85]	20-36 + 6 w 73 women	------	Ratio predictive and prognostic marker for PE. Confirms ratio as useful for both diagnosis and prognosis.
Kifle et al., 2022	[91]	24 + 0–37 + 0 w 370 women	-------	Models incorporating continuous values of sFlt-1 or the sFlt-1/PlGF ratio demonstrated superior predictive performance compared with models using PlGF alone or applying a fixed cutoff of 38 for the ratio. The study confirmed the clinical utility of sFlt-1/PlGF biomarkers for risk stratification in women with suspected preeclampsia, supporting their integration into predictive models to improve management and surveillance strategies.
Dathan-Stumpf et al., 2022	[86]	>33 w 283 women with hypertension	------------	The sFlt-1/PlGF ratio shows a positive correlation with the severity of placental dysfunction and an inverse relationship with time to delivery. The sFlt-1/PlGF ratio distinguishes between pregnancies with normal outcomes and those complicated by placental dysfunction.
Hughes et al., 2023	[92]	20 + 0–36 + 6 w 222 women	-----------	<37 w ratio ≤ 38 rules out PE in the subsequent week (NPV 96.2%) and ruled in PE within 4 weeks (PPV) 75%. In New Zealand, the predictive value of the sFlt-1/PlGF ratio aligns with international evidence. When applied in practice, it can help stratify risk in women with suspected preeclampsia and focus resources on those most vulnerable.

GA: gestation age, BP: blood pressure, SBP: systolic blood pressure, DBP: diastolic blood pressure, w: weeks, PE: preeclampsia, sFlt-1/PLGF: soluble fms-like tyrosine kinase-1/platelet growth factor, NPV: negative predictive value, PPV: positive predictive value.

## Data Availability

No new data were created or analyzed in this study. Data sharing is not applicable to this article.
